# Novel therapeutic targets for primary open-angle glaucoma identified through multicenter proteome-wide mendelian randomization

**DOI:** 10.3389/fphar.2024.1428472

**Published:** 2024-08-16

**Authors:** Weichen Yuan, Jun Li, Shang Gao, Wei Sun, Fangkun Zhao

**Affiliations:** ^1^ Department of Ophthalmology, The Fourth Affiliated Hospital of China Medical University, Shenyang, China; ^2^ Key Lens Research Laboratory of Liaoning Province, Shenyang, China; ^3^ Department of Ultrasonography, Shandong Provincial Hospital Affiliated to Shandong First Medical University, Jinan, China

**Keywords:** Mendelian randomization, drug targets, therapy, primary open angle glaucoma (POAG), plasma proteins

## Abstract

**Background:**

This study aimed to identify novel therapeutic targets for primary open-angle glaucoma (POAG).

**Methods:**

The summary-data-based Mendelian randomization (SMR) method was used to evaluate the genetic association between plasma proteins and POAG. Two sets of plasma protein quantitative trait loci (pQTLs) data considered exposures were obtained from the Icelandic Decoding Genetics Study and UK Biobank Pharma Proteomics Project. The summary-level genome-wide association studies data for POAG were extracted from the latest Round 10 release of the FinnGen consortium (8,530 cases and 391,275 controls) and the UK Biobank (4,737 cases and 458,196 controls). Colocalization analysis was used to screen out pQTLs that share the same variant with POAG as drug targets identified. The two-sample Mendelian randomization, reverse causality testing and phenotype scanning were performed to further validate the main findings. Protein-protein interaction, pathway enrichment analysis and druggability assessment were conducted to determine whether the identified plasma proteins have potential as drug targets.

**Results:**

After systematic analysis, this study identified eight circulating proteins as potential therapeutic targets for POAG. Three causal proteins with strong evidence of colocalization, ROBO1 (OR = 1.38, *p* = 1.48 × 10^−4^, PPH4 = 0.865), FOXO3 (OR = 0.35, *p* = 4.34 × 10^−3^, PPH4 = 0.796), ITIH3 (OR = 0.89, *p* = 2.76 × 10^−4^, PPH4 = 0.767), were considered tier one targets. Five proteins with medium support evidence of colocalization, NCR1 (OR = 1.25, *p* = 4.18 × 10^−4^, PPH4 = 0.682), NID1 (OR = 1.38, *p* = 1.54 × 10^−3^, PPH4 = 0.664), TIMP3 (OR = 0.91, *p* = 4.01 × 10^−5^, PPH4 = 0.659), SERPINF1 (OR = 0.81, *p* = 2.77 × 10^−4^, PPH4 = 0.59), OXT (OR = 1.17, *p* = 9.51 × 10^−4^, PPH4 = 0.526), were classified as tier two targets. Additional sensitivity analyses further validated the robustness and directionality of these findings. According to druggability assessment, Pimagedine, Resveratrol, Syringaresinol and Clozapine may potentially be important in the development of new anti-glaucoma agents.

**Conclusion:**

Our integrated study identified eight potential associated proteins for POAG. These proteins play important roles in neuroprotection, extracellular matrix regulation and oxidative stress. Therefore, they have promising potential as therapeutic targets to combat POAG.

## 1 Introduction

Glaucoma is the primary global cause of irreversible blindness, leading to progressive loss of visual field due to retinal ganglion cells (RGC) and optic nerve fibers damage, and is projected to affect 111.8 million people by 2040 (Tham et al., 2014). Primary open-angle glaucoma (POAG) is the predominant type of glaucoma, constituting 75%–90% of cases within the glaucoma patient population (Quigley and Broman, 2006). To date, the known risk factors for POAG mainly include age, diabetes, hypertension, intraocular pressure (IOP), race, and family genetic history (Choi and Kook, 2015). Among them, IOP is the most important and only modifiable risk factor. For every 1 mmHg in excess of normal IOP, the risk of glaucoma increases by 10% and 15% (de Voogd et al., 2005). Therefore, in clinical practice, various methods are used to reduce IOP to achieve the purpose of slowing the progression of vision loss caused by glaucoma, such as ophthalmic drug therapy, tube shunt implantation and trabeculectomy. However, about 10% of patients will develop resistance to topical eye drops, and postoperative fibrosis of subconjunctival and episcleral tissue will also lead to surgical failure (Doucette et al., 2018). At the same time, because RGC and axon loss are irreversible, most glaucoma patients will still continue to gradually lose their eyesight and are doomed to blindness. Therefore, there is still a need to find new drug targets, devices and other treatments, such as gene and cell therapy.

Proteins are important participants in the complex pathological process of POAG and valuable sources of potential biomarkers and drug targets. For example, stressed trabecular meshwork (TM) cells will release unusual collagens and fibronectins, accelerating the accumulation of extracellular matrix (ECM) proteins around the TM, further causing an increase IOP (Weinreb et al., 2014). Therefore, determining the relationship between protein expression level and diseases to identify potential biomarkers and drug targets is of great significance for the exploration of the disease diagnosis, treatment and pathogenesis. Previous studies usually applied methods such as liquid chromatography mass spectrometry to observe proteomic changes in the aqueous humor (AH) or plasma of glaucoma patients (Sharma et al., 2018; Beutgen et al., 2019). Shar et al. used this method to identify 33 proteins that were significantly altered in the AH of subjects with POAG^6^. These identified proteins may be involved in the potential pathogenesis of POAG through glycosylation, immune response, lipid metabolism and other pathways. However, these studies have limitations such as relatively small sample sizes and the inability to determine the directionality of relevant associations. In recent years, the rapid development of genome-wide association studies (GWAS) based on high-throughput DNA sequencing technology has provided a powerful method for identifying causative genetic variants and disease-causing proteins of common genetic diseases.

POAG is a complex genetic trait showing a significant genetic influence, with 60% of patients having a documented family history of the disease (Wolfs et al., 1998). More than 80 loci associated with POAG or its endophenotypes identified through GWAS to date (Youngblood et al., 2019). These loci linked POAG to several risk factors, such as myosin defects, *Cacna2d1* regulation in elevated IOP and retinal nerve fiber disease (Sharif, 2023). In most cases, however, it remains unclear which genes or proteins these variants regulate specifically to influence disease development. Recently, expression quantitative trait loci (eQTLs) and protein quantitative trait loci (pQTLs) identified by large-scale genomics and proteomics can help bridge the gap between the variants and diseases, elucidating the regulatory mechanisms of variants. Drug target Mendelian randomization (MR) can integrate these genetic data to determine causal relationships between genetically predicted gene or protein expression levels and genetic susceptibility to disease, thereby identifying potential drug targets (Fang et al., 2023). As we all know, randomized controlled trial (RCT) is the gold standard for evaluating interventions. However, due to their high cost and ethical restrictions, it is difficult to implement. MR can be compared to a “natural RCT”, in which alleles separated during meiosis and fertilization are randomly combined, and the presence or absence of alleles is similar to the drug groups and control group (Davey Smith, 2007).

Drugs developed based on genetic loci related to disease phenotypes have lower tendency of damaging side effects and are twice as likely to gain market approval compared to conventional drugs (Nelson et al., 2015). They are also more patient-centered and can become personalized drugs. Although gene therapy has the advantage that correcting a genetic defect can lead to a cure rather than just a treatment of symptoms, there are still limitations such as liver toxicity, carcinogenic issues, and blood disorders. Therefore, the screening of target proteins suitable for classical pharmacological therapy is more feasible. In this study, we performed multicenter MR, using the pQTLs data of plasma proteins as the exposures and the GWAS data of POAG as the outcome, to determine the protein-POAG pairs with genetic association. Colocalization analysis was additionally used to screen out proteins that could be potential drug targets. Multiple sensitivity analyses were performed to further verify our conclusions.

## 2 Methods

### 2.1 Study design

**FIGURE 1:**
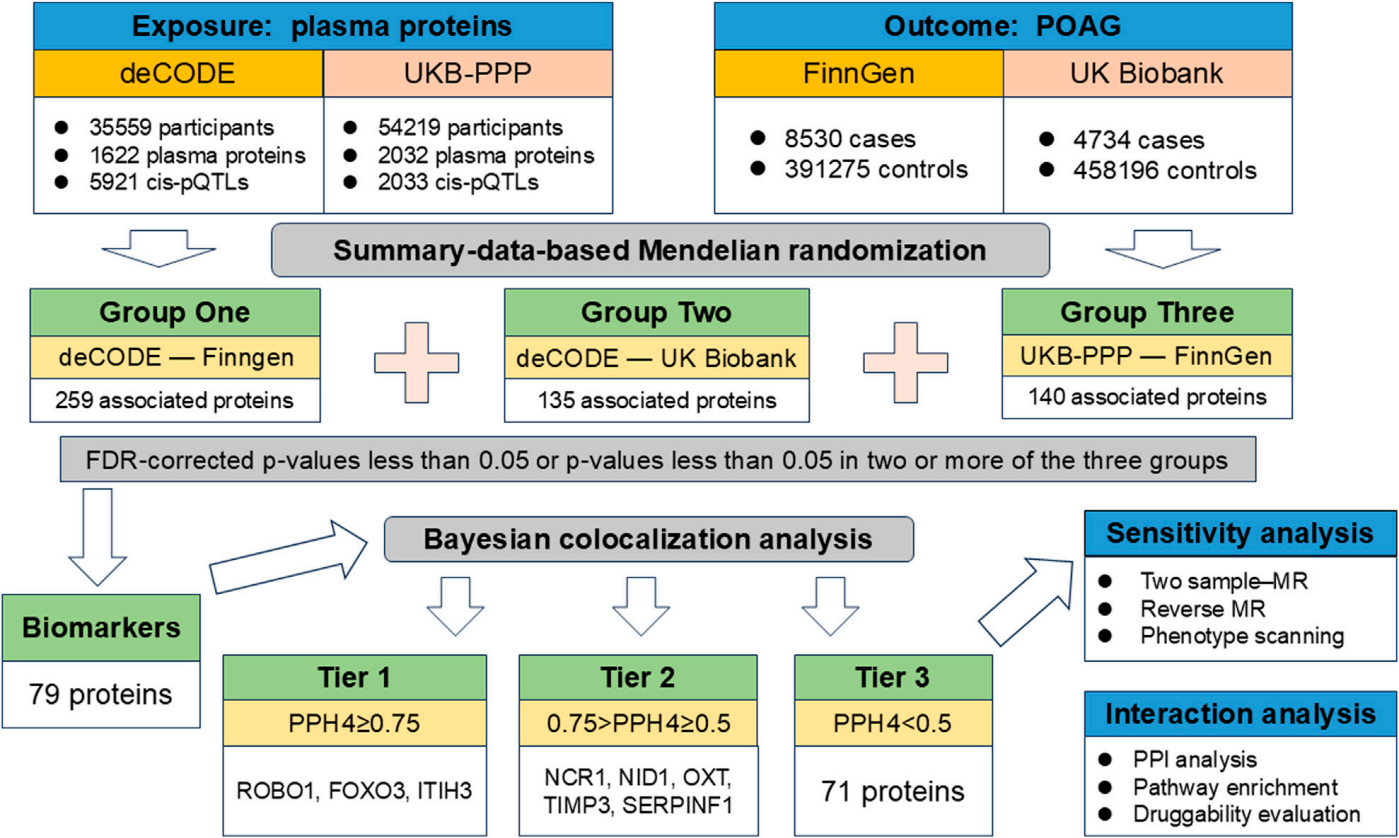
Flowchart of identifying causal plasma proteins for primary open-angle glaucoma by Mendelian randomization.

In order to reduce omissions and identify more potential drug targets, we have designed a unique multicenter analysis method, which is different from the previous MR analysis. First, we used the largest and latest two sets of plasma protein pQTLs data as exposures, and two sets of genome-wide association (GWAS) data for POAG as outcomes. Three groups of Summary-data-based MR (SMR) analyses were performed independently to identify proteins genetically related to POAG. Based on the results of the three groups of analyses, we considered pQTLs with FDR-corrected *p*-values less than 0.05 or pQTLs with *p*-values less than 0.05 in two or more of the three groups of SMR analyses as preliminary potential biomarkers. Next, colocalization analysis was used to screen out pQTLs that share the same variant with POAG as drug targets identified in this study. Then, we used two-sample MR, reverse causality testing and phenotype scanning to further validate the main findings. Finally, protein-protein interaction, pathway enrichment analysis and druggability assessment were performed to determine whether the identified plasma proteins have potential as drug targets. The flow chart of study design was showed in Figure 1.

### 2.2 Data sources

Two sets of plasma pQTL data considered exposures were obtained from the Icelandic Decoding Genetics Study (deCODE) and UK Biobank Pharma Proteomics Project (UKB-PPP). DeCODE reported the pQTL data of 4,907 plasma proteins measured in 35,559 Icelanders with SomaScan version four and UKB-PPP presented comprehensive pQTL mapping of 2,923 proteins measured from 54,219 UK Biobank participants using the Olink platform (Ferkingstad et al., 2021; Sun et al., 2023). In order to screen out instrumental variables (IVs) that meet MR assumptions, the pQTLs included in this study need to meet the following criteria: 1) were significant genome-wide association with plasma proteins (*p* < 5 × 10^−8^); 2) were *cis*-acting pQTLs; 3) showed independent association (linkage disequilibrium (LD) clumping *r* ^2^ < 0.001); 4) were not located in major histocompatibility complex (MHC) region; 5) were not palindromic single nucleotide polymorphisms (SNPs); 6) were not weak instrumental variables with F-statistic less than 10.

The summary-level GWAS data for POAG were extracted from the latest Round 10 release of the FinnGen consortium (8,530 cases and 391,275 controls) (https://r10.finngen.fi/) and the UK Biobank (4,737 cases and 458,196 controls), respectively. Assessment of diseases has been completed in the original study and does not require additional confirmation (Sudlow et al., 2015; Kurki et al., 2023).

The data used in this study were de-identified summary-level data obtained from publicly available databases and did not require additional ethical approval.

### 2.3 Statistical analysis

#### 2.3.1 SMR analysis

SMR is a method that integrates summary statistics from pQTL studies and GWAS under an MR framework to prioritize proteins whose expression levels may be causally related to the outcome trait (Wu et al., 2018). The SMR software package (V.1.03) was used to perform SMR analysis. The statistical findings are displayed using odds ratios (OR) and 95% confidence intervals (95% CI), with a significance threshold of *p*-value < 0.05. To prevent inaccurate positive results, a false discovery rate (FDR) correction was implemented.

#### 2.3.2 Bayesian colocalization analysis

In order to exclude causal relationships derived from LD, we performed Bayesian colocalization analysis for further screening for the proteins that met the threshold. The coloc.abf function in “coloc” package gives posterior probabilities for five hypotheses about whether SNP shares the same variants as exposure or outcome. The posterior probability of hypothesis 4 (PPH4) that both protein and POAG being associated with the region through shared variants greater than or equal to 0.75 was considered strong evidence of colocalization. A medium level of colocalization was defined as PPH4 being between 0.5 and 0.75 (Zhang et al., 2024). At the same time, proteins that showed strong evidence of colocalization (PPH ≥ 0.75) were categorized as tier one targets, those with moderate evidence (0.5 < PPH < 0.75) were classified as tier two targets, and the rest were grouped as tier three targets.

#### 2.3.3 Sensitivity analysis

We used two-sample MR as a complementary method to estimate the genetic associations between identified proteins levels and POAG. The Wald ratio method was utilized for proteins that had only one pQTL, whereas for proteins with two or more pQTLs, the inverse-variance-weighted (IVW) method was employed. In addition, Steiger filtering test and reverse two-sample MR were performed to determine the direction of causal correlation. The Steiger filtering method is based on the principle that a reliable IV should have a stronger impact on the exposure variable than on the outcome variable. When an IV meets this requirement, it is labeled as “TRUE”; otherwise, it is classified as “FALSE”. The criteria for screening IVs of POAG for reverse two-sample MR are the same as those of pQTL mentioned above. Finally, we used phenotype scanning to reveal whether the identified pQTL is associated with any known POAG risk factors to exclude potential pleiotropy (ldlink.nih.gov/?tab = ldtrait). All sensitivity analyses were performed in RStudio (version 4.3.1) and the significance level was defined as *p* < 0.05 corrected by FDR.

#### 2.3.4 Protein-protein interaction (PPI) and druggability evaluation

A STRING network was created using a PPI database to investigate the possible connections between the identified proteins (https://string-db.org/), with a minimum required interaction score of 0.4. In addition, in order to explore whether pathogenic proteins are enriched in certain pathways, we used Metascape online software to enrich suggestive pathogenic proteins (Zhou et al., 2019). Furthermore, we evaluated the druggability of the candidate target proteins by using DrugBank (Wishart et al., 2018) and DGldb (Freshour et al., 2021).

## 3 Results

### 3.1 Multicenter SMR analysis

After strict screening, 5921 *cis*-pQTLs for 1622 plasma proteins from deCODE and 2033 *cis*-pQTLs for 2032 plasma proteins from UKB-PPP were selected in our MR study as IVs. The detailed information of the screened pQTLs was shown in Supplementary Tables S1, S2. In order to screen drug targets more comprehensively, we used exposures and outcomes from multicenter datasets. In the Group One, using pQTLs from deCODE as the exposure and GWAS of POAG from FinnGen consortium as the outcome, the SMR analysis results showed that 310 pQTLs for 259 plasma proteins were genetically linked to POAG (Supplementary Table S3). In the Group Two, using pQTLs from deCODE as the exposure and GWAS of POAG from UK Biobank as the outcome, the SMR analysis results showed that 146 pQTLs for 135 plasma proteins were genetically linked to POAG (Supplementary Table S4). All the significance disappeared after FDR correction. In the Group Three, using pQTLs from UKB-PPP as the exposure and GWAS of POAG from FinnGen consortium as the outcome, the SMR analysis results showed that 140 pQTLs for 140 plasma proteins were genetically linked to POAG (Supplementary Table S5). According to study design, we considered pQTLs with FDR-corrected *p*-values less than 0.05 or pQTLs with *p*-values less than 0.05 in two or more groups as preliminary potential therapeutic targets for co-localization analysis. After sorting three groups of data, the results showed that 79 proteins meeting the above conditions were screened out for the next step of co-localization analysis (Supplementary Table S6).

### 3.2 Bayesian colocalization analysis

**FIGURE 2:**
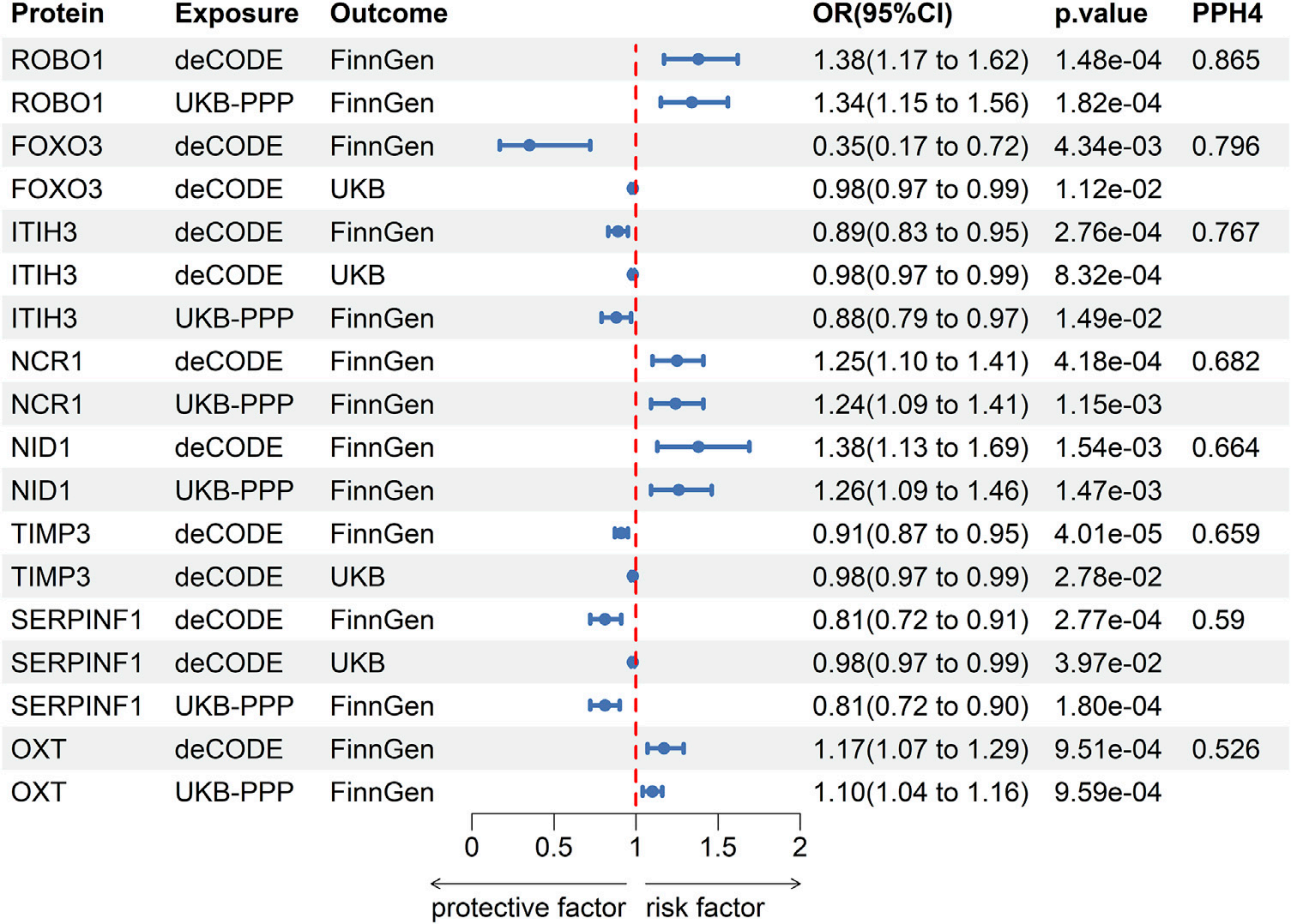
The results of SMR and Bayesian colocalization analysis of proteins identified as tier one or tier two.

The results of co-localization analysis for 79 proteins indicated that eight proteins shared the same genetic variants with POAG (Supplementary Table S6). The high support of colocalization evidence was observed between ROBO1 (OR = 1.38, *p* = 1.48 × 10^−4^, PPH4 = 0.865), FOXO3 (OR = 0.35, *p* = 4.34 × 10^−3^, PPH4 = 0.796), ITIH3 (OR = 0.89, *p* = 2.76 × 10^−4^, PPH4 = 0.767) and POAG, which were identified as tier 1. NCR1 (OR = 1.25, *p* = 4.18 × 10^−4^, PPH4 = 0.682), NID1 (OR = 1.38, *p* = 1.54 × 10^−3^, PPH4 = 0.664), TIMP3 (OR = 0.91, *p* = 4.01 × 10^−5^, PPH4 = 0.659), SERPINF1 (OR = 0.81, *p* = 2.77 × 10^−4^, PPH4 = 0.59) and OXT (OR = 1.17, *p* = 9.51 × 10^−4^, PPH4 = 0.526) were classified as tier two due to its moderate level of support from colocalization evidence. The remaining proteins with limited evidence of colocalization were ascertained as tier three targets. The results of SMR and Bayesian colocalization analysis of proteins identified as tier one or tier two were showed in Figure 2.

### 3.3 Sensitivity analysis

Two-sample MR analysis results based on IVW or Wald ratio verified the significance of the association between eight potential drug targets and POAG (Table 1). 49 SNPs of POAG was selected as IVs to evaluate the reverse causality (Supplementary Table S7). Reverse MR analysis and Steiger filtering did not reveal the existence of reverse causation (Table 1). After phenotype scanning, FOXO3 (rs3813498) was linked to intelligence and height, while ITIH3 (rs2071044) was associated with anxiety/tension. These proteins were not identified to be associated with risk factors for POAG.

**TABLE 1:** Summary of sensitivity analyses on eight potential drug targets.

Protein	Two-sample MR (OR, *p*-value)	Reverse MR (OR, *p*-value)	Steiger filtering
ROBO1	1.38 (1.17–1.62), 1.17 × 10^−4^	0.99 (0.97–1.02), 0.48	TRUE, 4.13 × 10^−2^
FOXO3	0.35 (0.19–0.67), 1.26 × 10^−3^	0.98 (0.96–1.01), 0.16	TRUE, 2.26 × 10^−38^
ITIH3	0.87 (0.82–0.93), 4.98 × 10^−6^	1.01 (0.99–1.03), 0.44	TRUE, 1.88 × 10^−100^
NCR1	1.25 (1.10–1.41), 3.88 × 10^−4^	1.00 (0.98–1.03), 0.66	TRUE, 2.28 × 10^−54^
NID1	1.29 (1.08–1.54), 5.33 × 10^−3^	1.01 (0.98–1.04), 0.47	TRUE, 2.82 × 10^−6^
TIMP3	0.91 (0.87–0.95), 3.99 × 10^−5^	1.00 (0.98–1.03), 0.83	TRUE, 7.77 × 10^−38^
SERPINF1	0.85 (0.77–0.93), 7.68 × 10^−4^	1.01 (0.98–1.03), 0.65	TRUE, 2.89 × 10^−160^
OXT	1.20 (1.04–1.37), 1.14 × 10^−2^	0.99 (0.97–1.02), 0.55	TRUE, 7.35 × 10^−38^

MR, Mendelian randomization; OR, odd ratio.

### 3.4 Protein-protein interaction (PPI) and druggability evaluation

**FIGURE 3:**
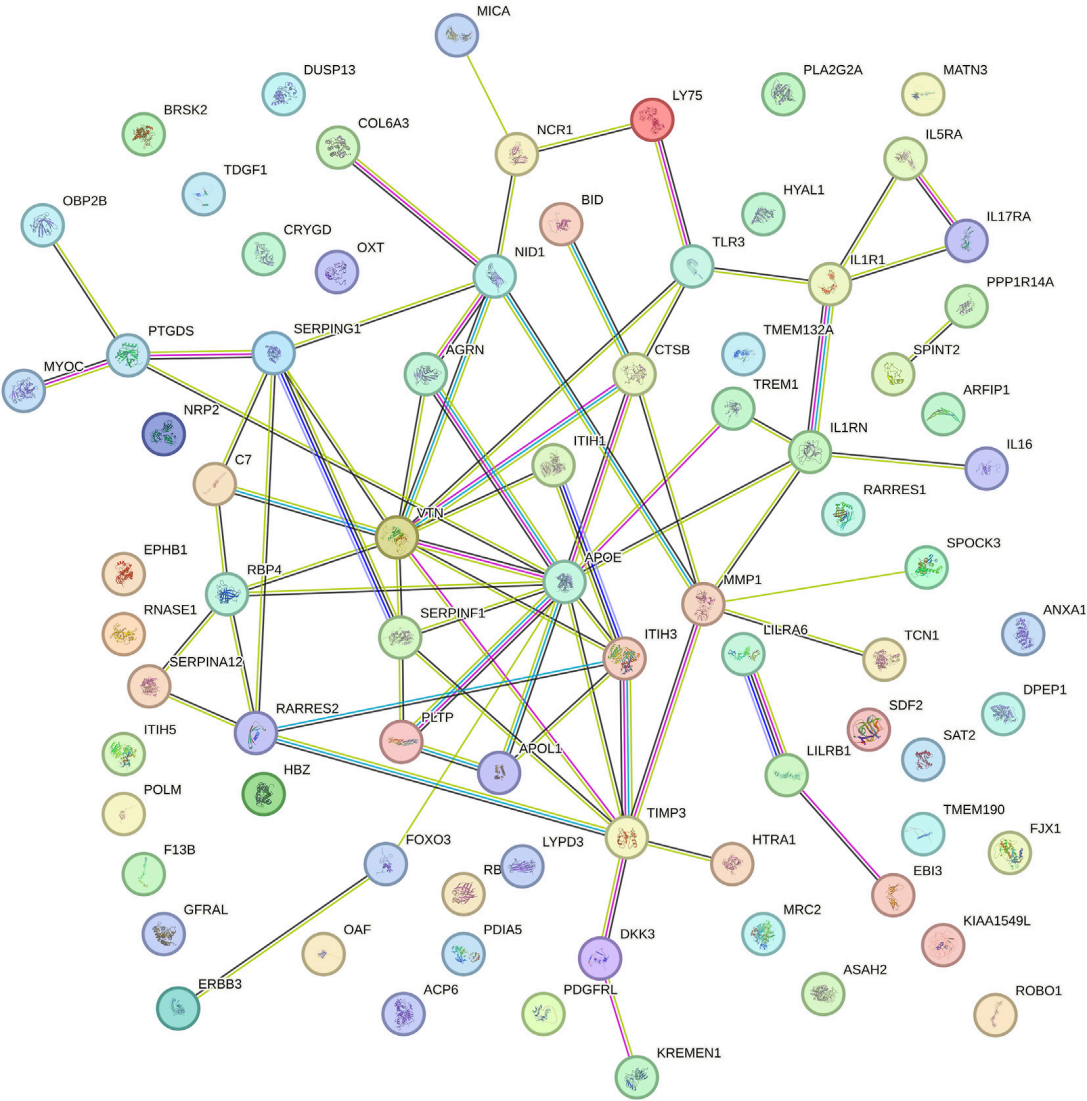
Protein-protein interaction network among the suggestive causal proteins.

**FIGURE 4:**
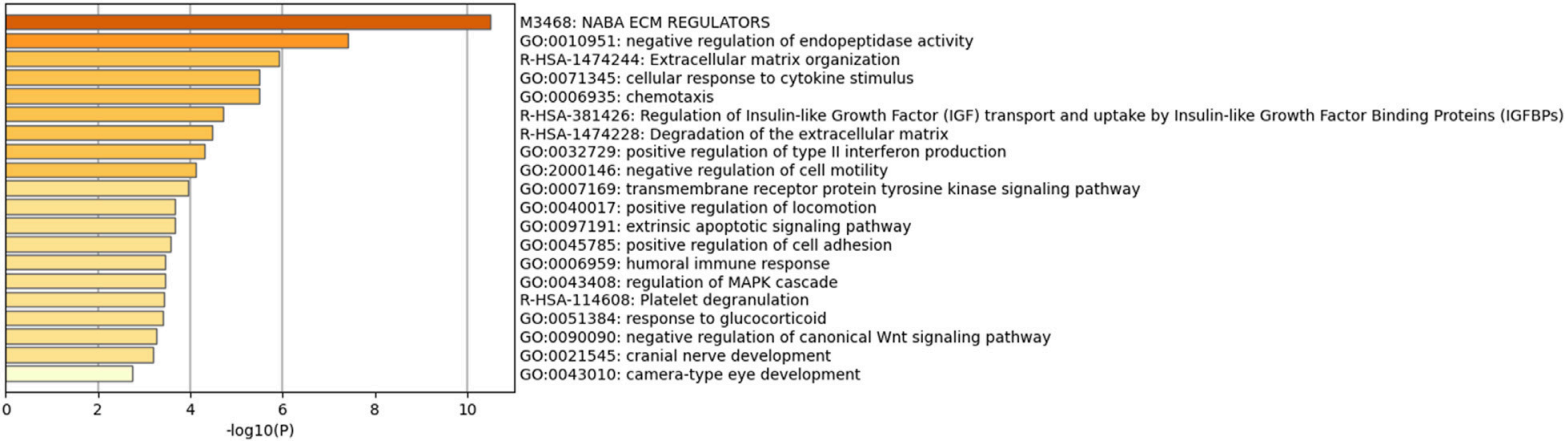
Pathway enrichment analysis among the suggestive causal proteins.

We performed a PPI analysis on the 79 identified plasma proteins responsible for causation. In Figure 3, the STRING network is illustrated with a medium model confidence of 0.400, highlighting 79 nodes and 65 edges representing interactions within the network. This exceeds the expected 16 interactions in a randomly selected set of proteins of the same size (*p*-value < 1 × 10^−16^). In the pathway enrichment analysis, we found that the suggested pathogenic proteins in plasma were significantly enriched in the “ECM regulation”, “negative regulation of endopeptidase activity” and “ECM organization” pathways (Figure 4).

In the evaluation of drug availability, six drugs that may change the disease were identified. Pimagedine targeting TIMP3 has been found to a beneficial effect in treating patients with diabetic nephropathy (Abdel-Rahman and Bolton, 2002). Resveratrol and Syringaresinol are targeted drugs for FOXO3. Clozapine targeting ITIH3 and OXT is a second-generation antipsychotic medication classified as atypical, which is utilized in the management of treatment-resistant schizophrenia and to reduce the risk of suicide in individuals with schizophrenia (Siskind et al., 2016). Zinc and Copper are targets drugs for ITIH3 and SERPINF1 respectively.

## 4 Discussion

To our knowledge, this is the first study to investigated the genetic causal association between the plasma proteins and POAG using multicenter proteome-wide MR analysis. After statistical analysis and data collation, the results showed that 79 plasma proteins are associated with the onset of POAG, which can be regarded as potential biomarkers. Moreover, Bayesian colocalization highlighted the causal effects of eight protein biomarkers. Collectively, three proteins (ROBO1, FOXO3, ITIH3) with strong convincing evidence (tier 1) and five proteins (NCR1, NID1, TIMP3, SERPINF1, OXT) with mild convincing evidence (tier 2) were identified as drug targets. Additional two-sample MR analysis and Steiger filtering test further validated the robustness and directionality of these findings. These potentially pathogenic proteins are significantly enriched in processes such as ECM regulation, negative regulation of endopeptidase activity. Druggability evaluation indicated Pimagedine, Resveratrol, Syringaresinol, Clozapine Zinc and Copper may be promising new drugs for the treatment of POAG.

ROBO1 (roundabout 1) is a member of the immunoglobulin gene superfamily ROBO1, and only showed limited distribution in normal adult tissues, mainly in brain, kidney and eye (Huang et al., 2010). ROBO1 regulates the correct targeting of retinal ganglion cell axons throughout the visual projection (Plachez et al., 2008). Multiple previous experimental studies have also shown that slit-ROBO signaling promoted the formation of ocular neovascularization to alter the pathophysiological progression of proliferative retinal diseases (Huang et al., 2010; Rama et al., 2015). In addition, Jun et al. (2011) reported that distinct *ROBO1* variants may influence the risk of wet and dry age-related macular degeneration (AMD) (Jun et al., 2011). However, there are currently no publications reporting the association between ROBO1 and the onset of glaucoma. Our study provided the first strong evidence that elevated ROBO1 expression levels increase genetic susceptibility to POAG. Based on these findings, the development of small molecule inhibitors of ROBO1 is of great significance for the treatment of POAG and other eye diseases.

Forkhead box O3 (FOXO3) proteins are considered to be ideal therapeutic targets because of their overall ability to control cell proliferation, metabolism and antioxidant defense (Bernardo et al., 2023). Oxidation-induced TM damage can increase IOP and promote the occurrence of glaucoma. RGC apoptosis is also related to oxidative stress. Multiple studies have shown that oxidative stress markers in the AH of patients with glaucoma are significantly altered (Benoist d'Azy et al., 2016). Experimental studies show that Nipradilol and timolol increase the expression of FOXO3a and peroxiredoxin 2, leading to the protection of trabecular meshwork cells from oxidative stress (Miyamoto et al., 2009). Consistent with these results, we further expanded the evidence and established that the elevated FOXO3 protein levels are a protective factor against POAG. Resveratrol, an activator of FOXO3, is thought to have anti-inflammatory, antioxidant, anti-glycation, neuroprotective properties and has a beneficial effect on ocular tissue (Franco et al., 2014). In the pathophysiological process of glaucoma, Resveratrol can effectively reduce the production of ROS to reduce the damage of trabecular meshwork cells caused by oxidative stress and reduce intraocular pressure. At the same time, the use of Resveratrol can also play a role in neuroprotection and improve the survival rate of RGCs(Bryl et al., 2022). Another novel drug, Syringaresinol, which targets FOXO3, has anti-tumor and antioxidant activities. Syringaresinol reduces oxidative stress by activating the Nrf2 antioxidant pathway, improving retinal microvascular damage by inhibiting HIF-1/VEGF and thus easing the early progression of diabetic retinopathy (Liu et al., 2024). Therefore, considering the possibility of using these natural compounds to treat stress oxidative diseases such as glaucoma.

ITIH3 is the inter-alpha-trypsin inhibitor heavy chain (ITIH) protein involved in the stabilization of the extracellular matrix (Bost et al., 1998). One of the main regulatory sites of AH drainage is thought to be located in the ECM of the juxtacanalicular tissue. The overall level of ECM molecules will affect the structure and tissue of outflow resistance. Current evidence suggests that slight changes in the composition of ECM are more likely to affect AH outflow by changing the compliance of TM or the contractile properties of TM cells (Keller and Peters, 2022). Polymorphisms in ITIH3 has been implicated in several diseases, including schizophrenia and major depressive disorder (Xie et al., 2020). So far, no genetic association of ITIH3 polymorphisms with POAG has been reported. Based on the results of druggability evaluation, Zinc and Clozapine are drugs targeting ITIH3. Zinc is generally considered an antioxidant factor and plays a role in the pathophysiology of glaucoma (Ugarte and Osborne, 2001). Zinc may be involved in light-induced retinal damage, and reduced zinc levels may increase oxidative stress, which is thought to contribute to pseudoexfoliation glaucoma in cataract patients (Yildirim et al., 2007). Previous studies have shown that clozapine can relax bovine retinal veins and increase blood flow in the iris, retina, and choroid (Boussery et al., 2005). At the same time, local instillation in the eye can reduce the IOP of rabbits with high intraocular pressure to a certain extent (Boussery et al., 2005). These conclusions imply that clozapine May help treat glaucoma. However, there is a lack of more in-depth research.

Tissue inhibitor of metalloproteinase 3 (TIMP3) is a well-known matrix metalloproteinase inhibitor. The balance between the two affects a variety of physiological functions, including extracellular matrix remodeling, apoptosis, cell migration, and proliferation (Joe et al., 2017). Overexpression of TIMP3 can lead to dynamic imbalance between ECM synthesis and degradation, affect AH excretion, further increase IOP and aggravate POAG damage (Ji and Jia, 2019). Pimagedine targeting TIMP3 acts as an inhibitor of advanced glycation end products and is effective in managing diabetic nephropathy when used on its own or in conjunction with other treatments (Corbett et al., 1992). Animal experiments by Neufeld et al. showed that rats treated with Pimagedine lost fewer RGC during the increase in IOP than the control group (Neufeld et al., 1999). With this neuroprotective effect, aminoguanidine may become a novel drug for the treatment of glaucoma patients.

The SERPINF1 (Serpin Family F Member 1) gene encodes pigment epithelium derived factor (PEDF). PEDF is a powerful protein with antiangiogenic, neurotrophic, and neuroprotective properties and has the ability to shield neurons from various harmful factors (Pang et al., 2007). It is reported that SERPINF1 is involved in the migration and invasion of ECM remodeling in gastric cancer (Lee et al., 2022). Copper (targeting SERPINF1) is generally considered to have an antioxidant effect in human metabolism. The concentration of copper ions in the AH of rabbits treated with steroids was significantly lower than that of the control. It was inferred that steroid hormones may cause an increase in IOP by interfering with the homeostasis of copper ions, which may explain the protective role of Cu in the pathogenesis of OAG (Iqbal et al., 2002).

NCR1, or natural cytotoxicity triggering receptor 1, is expected to play a role in cellular defense response, controlling the cytotoxicity of natural killer cells, and transmitting signals. NCR1 has been linked to a variety of diseases, including viral infections, cancer and autoimmune diseases. NCR1 has been implicated in natural killer cell recognition and elimination of infected cells (Mandelboim and Porgador, 2001).

NID1, also known as nidogen 1, belongs to the nidogen family of proteins and is an essential component of the basement membrane. NID1 is involved in cell interactions with the extracellular matrix and may play a role in various biological processes such as cell adhesion, migration, and differentiation. NID1 is present in all tissues related to AH turnover. The anterior segments of NID1 knockout mice showed mild but significant morphological changes pointing to the importance of this protein. However, no difference in intraocular pressure was observed (May, 2012).

Oxytocin (OXT) is a pleiotropic, peptide hormone with broad implications for general health, adaptation, development, reproduction, and social behavior (Carter et al., 2020). Oxytocin has the ability to act as a stress-regulating compound, an anti-inflammatory agent, and an antioxidant, providing a shield against challenges or trauma. Oxytocin influences the autonomic nervous system and the immune system (Carter et al., 2020). The OXT gene has been implicated in several disorders, including autism spectrum disorder, social anxiety disorder, and schizophrenia. Studies have shown that variations in the gene may affect oxytocin levels and function, leading to altered social behavior and cognition (Jacob et al., 2007). At present, direct evidence on NCR1, NID1, OXT and POAG risk has not been reported, and further epidemiological and experimental studies are needed to confirm our findings.

The novelty of this study lies in its unique study design. Traditional drug target MR uses proteins that remain significant after multiple correction in the main analysis as drug targets, and performs external validation, co-localization analysis, sensitivity analysis, etc. to verify the main findings. However, the identifying of drug targets is not simply a matter of determining cause and effect. Using FDR correction is easy to miss some potential drug targets, which has major limitations. Our study has improved this problem to a certain extent. However, it is essential to acknowledge several limitations. Firstly, the pQTL data of the proteins we selected were derived from plasma and may not fully reflect the specific changes in retinal tissue or AH. Future studies should use ocular tissue-derived proteins to further explore drug targets. Secondly, most proteins have only one corresponding pQTL, which may reduce statistical power and prevent heterogeneity analysis and pleiotropy detection. Thirdly, the biological relevance of most of the identified drug targets to glaucoma is unclear, and we can only infer their role in the pathological progression of glaucoma based on their properties. Further research is needed to elucidate the mechanisms of these candidate proteins in POAG. Finally, caution is needed when generalizing our findings because pQTL and GWAS data are mainly derived from individuals of European ancestry.

## 5 Conclusion

In conclusion, our proteome-wide MR analysis highlighted the genetic link between the plasma proteins ROBO1, FOXO3, ITIH3, NCR1, NID1, TIMP3, SERPINF1, OXT and the risk of POAG. These proteins play important roles in neuroprotection, extracellular matrix regulation and oxidative stress. Therefore, they have promising potential as therapeutic targets to combat POAG. Additional experimental and clinical research is necessary to assess the usefulness and effectiveness of these potential options and confirm the current results.

## Data Availability

The original contributions presented in the study are included in the article/Supplementary Material, further inquiries can be directed to the corresponding authors.
